# Income and wealth as correlates of socioeconomic disparity in dentist visits among adults aged 20 years and over in the United States, 2011–2014

**DOI:** 10.1186/s12903-018-0613-4

**Published:** 2018-08-23

**Authors:** Alexander Kailembo, Carlos Quiñonez, Gabriela V. Lopez Mitnik, Jane A. Weintraub, Jennifer Stewart Williams, Raman Preet, Timothy Iafolla, Bruce A. Dye

**Affiliations:** 1 National Institutes of Health, National Institute of Dental and Craniofacial Research, Bethesda, USA; 2 University of Toronto, Toronto, Canada; 3 University of North Carolina, Chapel Hill, USA; 4 Umeå University, Umea, Sweden; 5 Research Centre for Generational Health and Ageing, University of Newcastle, Callaghan, Australia

**Keywords:** Disparities, Inequalities, Socioeconomic position, Income, Wealth, Dental utilization, Dental visits

## Abstract

**Background:**

Most studies in the United States (US) have used income and education as socioeconomic indicators but there is limited information on other indicators, such as wealth. We aimed to assess how two socioeconomic status measures, income and wealth, compare as correlates of socioeconomic disparity in dentist visits among adults in the US.

**Methods:**

Data from the National Health and Nutrition Examination Survey (NHANES) 2011–2014 were used to calculate self-reported dental visit prevalence for adults aged 20 years and over living in the US. Prevalence ratios using Poisson regressions were conducted separately with income and wealth as independent variables. The dependent variable was not having a dentist visit in the past 12 months. Covariates included sociodemographic factors and untreated dental caries. Parsimonious models, including only statistically significant (*p* < 0.05) covariates, were derived. The Akaike Information Criterion (AIC) measured the relative statistical quality of the income and wealth models. Analyses were additionally stratified by race/ethnicity in response to statistically significant interactions.

**Results:**

The prevalence of not having a dentist visit in the past 12 months among adults aged 20 years and over was 39%. Prevalence was highest in the poorest (58%) and lowest wealth (57%) groups. In the parsimonious models, adults in the poorest and lowest wealth groups were close to twice as likely to not have a dentist visit (RR 1.69; 95%CI: 1.51–1.90) and (RR 1.68; 95%CI: 1.52–1.85) respectively. In the income model the risk of not having a dentist visit were 16% higher in the age group 20–44 years compared with the 65+ year age group (RR 1.16; 95%CI: 1.04–1.30) but age was not statistically significant in the wealth model. The AIC scores were lower (better) for the income model. After stratifying by race/ethnicity, age remained a significant indicator for dentist visits for non-Hispanic whites, blacks, and Asians whereas age was not associated with dentist visits in the wealth model.

**Conclusions:**

Income and wealth are both indicators of socioeconomic disparities in dentist visits in the US, but both do not have the same impact in some populations in the US.

**Electronic supplementary material:**

The online version of this article (10.1186/s12903-018-0613-4) contains supplementary material, which is available to authorized users.

## Background

Despite general improvements in oral health status due to advancement in social and living conditions, oral diseases remain among the most prevalent human diseases globally and a major public health problem. The 2015 Global Burden of Disease Study estimated that oral conditions (untreated dental caries, chronic periodontitis, and edentulism) ranked among the top ten health conditions, affecting 3.5 billion people worldwide [1]. The profile of oral diseases is not homogeneous between or within countries; the burden is substantially higher among poorer and disadvantaged populations in both high- and low-income countries, including the United States (US) [2–4]. In the US, people of lower socioeconomic position have a higher burden of oral diseases compared with those who are socioeconomically better off, and these disparities also apply to issues of access to oral health services. A study among US adults reported that people living in poverty and those with the least education had fewer dentist visits compared with more affluent and educated individuals [5].

Having a dentist visit in the past 12 months is one of the 17 Healthy People (HP) 2020 oral health objectives in the US [6]. The HP 2020 initiative contains health promotion and disease prevention national goals and objectives set by the US Department of Health and Human Services (HHS) for improving the health of all Americans. There are 12 Leading Health Indicators in HP 2020, and oral health is represented by the objective “to increase the proportion of children, adolescents, and adults who used the oral health care system in the past year.” Utilizing dental care in the past 12 months is correlated to higher levels of dental health satisfaction and overall quality of life [7]. Furthermore, differences in access to dental care still exist and a major reason for not having a dentist visit is financial circumstances [8]. For example, dental care utilization in the past 12 months among poor adults in the US was around 20% compared to approximately 50% among high income adults in 2014 [9].

Most studies in the US have used income [10, 11] and education [5, 12] as socioeconomic indicators to assess the association between lower socioeconomic position and lower rates of dentist visits, but there is limited information on other socioeconomic indicators, such as wealth, which measures accumulated assets rather than income alone. Yet there are arguments that health studies should include wealth as a socioeconomic indicator [13]. The wealth index has been widely used as a proxy of socioeconomic position in studies conducted in low- and middle-income settings. Based on methodology developed by Filmer and Pritchett [14], the index combines data on durable assets (car, refrigerator, and television), housing characteristics (dwelling floor and roof material), and access to services (drinking water source and electricity supply) and uses Principal Component Analysis to generate weights for household assets [14, 15]. However, in the US setting, where most households have durable assets and access to electricity and water supplies, it may be more appropriate to measure wealth differently, by combining income and assets such as cars, homes, savings, and stocks.

Even though income and wealth are positively correlated, they measure different things. For example, older adults may have little income but may have accumulated substantial wealth, through a combination of a lifetime of work and/or inheritance from ancestors. Recent immigrants and racial minorities, even those with high incomes, may be less likely to have significant familial or inherited wealth [16]. Income and occupation status among retired people lose their significance as measures of socioeconomic position and wealth becomes more important [17, 18]. Another example of the difference between income and wealth has been shown in studies using home ownership as a measure of wealth. Home owners may have more income to spend on health care than non-home owners because non-home owners still have to make rent payments [19]. Most recently, wealth rather than income was reported to be more sensitive as an indicator of socioeconomic disparity in health-related outcomes [13]. However, the best choice of socioeconomic measure will depend upon the purpose of the analysis and the policy context.

The purpose of this study is to improve understanding of income and wealth as correlates of socioeconomic disparity in dentist visits among adults in the US so that policy makers may be better informed about targeting interventions to improve access to dental care. The objective is to assess how two socioeconomic status measures, income and wealth, compare as correlates of socioeconomic disparity in dentist visits among adults in the US.

## Methods

### Data source

Data from the 2011–2014 National Health and Nutrition Examination Survey (NHANES) were used for this study. NHANES is a cross-sectional survey conducted by the National Center for Health Statistics (NCHS) of the Centers for Disease Control and Prevention (CDC) that uses a stratified, multistage sampling design to obtain a representative probability sample of non-institutionalized, civilian population of all ages in the US. NHANES data are used to assess the health, nutritional status and health behaviors of the eligible population using self-reported responses, standardized physical examinations and laboratory tests. The NHANES protocol was developed and reviewed to be in compliance with the HHS Policy for Protection of Human Research Subjects (45 CFR part 46). The protocol was approved by the NCHS Research Ethical Review Board and underwent annual review. Sample persons were informed of the survey process and their rights as a participant by interviewers and by written materials. Written informed consent was freely obtained from each individual participant. Only de-identified observations were used and presented in aggregated summary form. The current study combined the 2011–2012 and 2013–2014 NHANES cycles to derive a study sample covering the 2011–2014 period. Data from both cycles were collected via in-home interviews, with health examinations and laboratory tests conducted in mobile examination centers (MEC). Data for this analysis were obtained from responses to the home interviews and results from the dental examinations. The home interviews used a structured questionnaire that assessed demographic and socioeconomic characteristics and various health-related issues, including oral health. The dental examinations were conducted by trained dentists in the MEC on all eligible participants aged 1 year and older. The 2011–2012 and 2013–2014 NHANES data sets followed the same protocols and are in the public domain, analyzed using only de-identified observations and presented in aggregated summary form. Further details on NHANES survey sample design are provided elsewhere [20].

### Study population

The available data set of the 2011–2014 NHANES comprised 19,931 participants of all ages. From this study population, 8602 participants who were younger than 20 years were excluded. Additionally, all participants aged 20 years and over with no dental examination information (422) and those with missing observations (1661) were excluded. A complete flowchart of participants is available in Additional file 1.

Published response rates were 73% and 70% respectively for the interview and examination samples in the 2011–2012 cycle and 71% and 69% respectively for the 2013–2014 cycle [21, 22]. To increase the precision of estimates, NHANES oversamples some population subgroups. For NHANES 2011–2014, the primary change was the addition of an oversample of Asian persons. Oversampling was also carried out for Hispanic, non-Hispanic black, low-income white persons, and older white adults aged 80 years and over [20]. Sampling weights used in this data analysis are provided in the data files which are in public domain.

### Variables

The outcome was derived from the question “About how long has it been since you last visited a dentist?” Participants who responded “6 months or less” or “more than 6 months but not more than 12 months” were defined as having a dentist visit in the past 12 months. Participants who responded “more than 12 months” or “never have been” were classified as not having a dentist visit in the past 12 months.

The income variable was based on the poverty income ratio (PIR) calculated by dividing family income to the poverty level threshold specific to family size and survey year. The PIR followed the HHS federal poverty guidelines (FPG) which are issued each year, in the Federal Register, for determining financial eligibility for certain federal programs such as Head Start, Supplemental Nutrition Assistance Program (SNAP) and the National School Lunch Programs. Additional information can be located at http://aspe.hhs.gov/poverty/11poverty.shtml. Ratios below 1.00 indicate that family income is below the official definition of poverty and ratios of 1.00 or greater indicate family income at or above the poverty level. Income was categorized into four PIR groups [23]: PIR greater or equal to 3.00 (high income); PIR greater or equal to 2.00 and less than 3.00 (middle income); PIR greater or equal to 1.00 and less than 2.00 (near poor); and PIR less than 1.00 (poor).

The wealth variable was constructed using a combination of family monthly income and home ownership variables. We chose to use wealth as a socioeconomic variable in addition to income because some authors have argued that income in combination with assets such as housing is a better indicator of health than income alone [24]. Wealth was categorized into three groups – high, middle and low wealth groups. High wealth corresponds to participants in households with more than $2900 USD of monthly income and who are home owners. Middle wealth corresponds to participants with either more than $2900 USD of monthly income and who are non-home owners or are home owners with less than $2900 USD of monthly income. Low wealth corresponds to participants in households with less than $2900 USD of monthly income and who are non-home owners. The choice of $2900 USD cut-off point was influenced by NHANES data reporting. The monthly income variable made available to the public is not presented as a continuous variable but rather as a range value in dollars. For example, $0 - $399 coded as 1, $400 - $799 coded as 2, $800 - $1249 coded as 3 etc. Therefore, our final binary categorization ensured participants’ income is as close to the US median value as possible and are equally divided.

Other variables used in the analyses were chosen based on Andersen’s behavioral model of health service use [25]. Predisposing factors included sex, age, race/ethnicity, country of birth, marital status, education, and smoking status. Age was categorized as 20–44 years, 45–64 years, and 65 years and over. Race/ethnicity was recoded as non-Hispanic white, Hispanic, non-Hispanic black, and non-Hispanic Asian. Country of birth was dichotomized as born in the US or born in another country. Marital status was recoded as married or cohabiting, widowed or separated, and never married. Educational attainment was classified as having more than 12 years, 12 years, and less than 12 years of school. Smoking status was categorized as current, former, and never smokers. Job status was an enabling factor being expressed as having a job versus not having a job. Need factors were self-rated oral health and the presence of untreated caries. Self-rated oral health was based on the question “Overall, how would you rate the health of your teeth and gums?” Participants were defined as satisfied if they responded excellent, very good or good, and not satisfied if they responded fair or poor. Presence of untreated dental caries (yes/no) was derived from the NHANES clinical dental examination.

### Statistical analysis

Only records with complete data on all study variables were analyzed. The analyses were carried out using STATA 13 software (StataCorp, 2013). Sample weights and survey design variables were used to account for unequal probability of participant selection, nonresponse, and sampling error. Absolute numbers, weighted percentages, and standard errors (SE) were estimated to assess the prevalence of not having a dentist visit in the past 12 months. Differences between percentages were calculated by using two-sided t-tests at the α = 0.05 level following recommended NHANES analytical and tutorial guidelines. Because education is associated with income and wealth in some populations, we assessed for collinearity between income and wealth with education and found weak correlation: 0.39 (income) and 0.25 (wealth).

Unadjusted (univariable) and parsimonious Poisson regression models were derived separately for income and wealth using generalized linear models with long link and Poisson distribution. The models described associations between income, wealth, socio-demographic factors, health behaviors and need factors and not having a dentist visit. The Poisson regression models were first adjusted for age, sex, race/ethnicity, country of birth, marital status, education, smoking status, job status, self-rated oral health and presence of untreated caries. The criterion for inclusion in the parsimonious models was *p* < 0.05 in the full adjusted models. Associations are presented as prevalence ratios (RRs) with 95% confidence intervals (CIs). The income and wealth models were compared using the Akaike Information Criterion (AIC) as a means of model selection. The AIC estimates the relative information lost when a given model is used to approximate reality. It selects the better model given a set of available data. A preferred model is the one with a minimum AIC valuee [26, 27]. Due to significant interactions by race/ethnicity, the results in the parsimonious models were stratified by race/ethnicity.

## Results

The study sample of adults aged 20 years and over in the US was 9246. The weighted prevalence of selected demographic and socioeconomic characteristics are presented in Table 1. Almost half of the adults were aged 20–44 years and about 18% were 65 years and over. The proportion of females to males was just above 50%. Just over two-thirds of the adults were non-Hispanic white (69%), approximately 12% were non-Hispanic black and 5% were non-Hispanic Asian. Almost two-thirds of adults had more than 12 years of education (64%), while only 15% had less than 12 years of education. Almost half of the adults aged 20 years and over lived in high income households, whereas 17% lived in poor households (below the federal poverty level). Almost half (48%) were in the high wealth group while 22% belonged to the low wealth group.

**Table 1:** Prevalence of not having a dentist visit in the past 12 months among adults aged 20 years and over, NHANES, 2011–2014^d^

Characteristic		Total study participants	Participants not having dentist visit	
		*n* (%)^a^	*n* ^b^	% (SE)^c^
	Total	9246	4171	38.8 (1.3)
Age groups	20–44 years	4057 (45.7)	1942	42.9 (1.7)^*^
	45–64 years	3149 (36.5)	1340	35.6 (1.7)^*^
	65+ years	2040 (17.8)	889	34.7 (1.7)^*^
Sex	Female	4792 (52.1)	1985	36.1 (1.5)^*^
	Male	4454 (47.8)	2186	41.7 (1.5)^*^
Race/ethnicity	Non-Hispanic White	3938 (69.1)	1604	34.0 (1.4)^*^
	Hispanic	1931 (14.1)	1011	53.9 (2.2)^*^
	Non-Hispanic Black	2190 (11.6)	1093	49.2 (1.8)^*^
	Non-Hispanic Asian	1187 (5.2)	463	38.3 (2.1)
Country of birth	Born in the United States	6598 (83.3)	2941	37.3 (1.4)^*^
	Born in other countries	2648 (16.7)	1230	46.2 (1.7)^*^
Marital status	Married/cohabiting	5348 (62.1)	2226	35.0 (1.5)^*^
	Widowed/separated	2059 (18.8)	1047	46.6 (1.7)^*^
	Never married	1839 (19.1)	898	43.4 (1.6)^*^
Education	More than 12 years	5234 (64.4)	1884	30.9 (1.2)^*^
	12 years	1995 (20.5)	1034	47.4 (1.6)^*^
	Less than 12 years	2017 (15.1)	1253	60.6 (1.9)^*^
Income	High income	3415 (48.2)	905	23.8 (1.3)^*^
	Middle income	1222 (14.1)	556	41.9 (2.5)
	Near poor	2412 (21.0)	1379	55.9 (1.5)^*^
	Poor	2197 (16.7)	1331	58.0 (2.1)^*^
Wealth	High	3441 (47.6)	1002	24.8 (1.3)^*^
	Middle	3088 (30.1)	1547	47.3 (1.7)^*^
	Low	2717 (22.3)	1622	57.1 (1.6)^*^
Job status	With a job	5068 (61.9)	2167	37.7 (1.4)^*^
	Without a job	4178 (38.1)	2004	40.5 (1.5)^*^
Smoking status	Never smoker	5230 (56.4)	2121	34.6 (1.8)^*^
	Former smoker	2148 (24.0)	949	36.1 (1.5)^*^
	Current smoker	1868 (19.6)	1101	54.3 (1.5)^*^
Self-rated oral health status	Satisfied	6231 (72.7)	2335	31.1 (1.4)^*^
	Not satisfied	3015 (27.3)	1836	59.3 (1.5)^*^
Untreated dental caries	No	6579 (75.4)	2528	32.0 (1.3)^*^
	Yes	2667 (24.6)	1643	59.5 (1.7)^*^
Dentist visit in the past 12 months	Yes	5075 (61.2)		
	No	4171 (38.8)		

^*^p < 0.05 (t-statistic)

^a^n (%) Number and weighted percent of entire study participants

^b^n Number of respondents not having a dentist visit in the past 12 months

^c^%(SE) Weighted percent and Standard Error of participants not having a dentist visit in the past 12 months

^d^Data source – National Health and Nutrition Examination Survey (NHANES), 2011–2014

Table 1 also shows the prevalence of not having a dentist visit in the past 12 months among adults aged 20 years and over by sociodemographic characteristics. The overall prevalence was 39%, with a significant difference between participants who did or did not have a dentist visit. This difference held true for all groups except for the non-Hispanic Asian and middle income groups. An income gradient was observed whereby participants in the poorest group had the highest prevalence (58%) and those in the highest income group had the lowest prevalence (24%) of not having a dentist visit. A wealth gradient was also observed. The prevalence was highest in the lowest wealth group (57%) and lowest in the highest wealth group (25%). Not having a dentist visit was more prevalent among adults aged 20–44 years, males, Hispanics, those with the least education, current smokers, those who reported poor oral health, and those with the presence of untreated dental caries.

The income and wealth regression models are presented in Table 2. In the univariable model, adults in the poor (RR = 2.44 95% CI 2.17–2.73) and low wealth (RR = 2.30 95% CI 2.05–2.58) groups were over two times more likely to not have a dentist visit in the past 12 months compared with those with high income and high wealth. Participants aged 20–44 years were 24% (RR = 1.24 95% CI 1.10–1.38) more likely to not have a dentist visit compared with those aged 65 years and over. However, there were no differences between adults aged 45–64 years and those 65 years and over. For adults aged 20 and above in the US, the following factors were associated with not having a dental visit in the past 12 months: being male; Hispanic; non-Hispanic black; born outside the US; widowed or separated; never married; least educated; a current smoker; not satisfied with oral health; having no job; and with untreated dental caries.

**Table 2:** Income, wealth and other factors associated with not having a dentist visit in the past 12 months among adults aged 20 years and over in the United States^d^

		Unadjusted Model	Pars Income Model^a^	Pars Wealth Model^b^
		RR (CI)	RR (CI)	RR (CI)
Age groups	20–44 years	**1.24 (1.10–1.38)**	**1.16 (1.04–1.30)**	ns
	45–64 years	1.03 (0.93–1.13)	1.02 (0.94–1.12)	ns
	65+ years^c^	1.00	1.00	
Sex	Female^c^	1.00	1.00	1.00
	Male	**1.15 (1.07–1.24)**	**1.14 (1.06–1.22)**	**1.13 (1.05–1.21)**
Race/ethnicity	Non-Hispanic White^c^	1.00	1.00	1.00
	Total Hispanics	**1.58 (1.42–1.77)**	**1.16 (1.07–1.25)**	**1.20 (1.10–1.76)**
	Non-Hispanic Black	**1.45 (1.31–1.60)**	**1.12 (1.05–1.20)**	**1.12 (1.04–1.21)**
	Non-Hispanic Asian	1.13 (0.97–1.30)	**1.22 (1.09–1.37)**	**1.20 (1.07–1.35)**
Country of birth	Born in the United States^c^	1.00		
	Born in other countries	**1.24 (1.14–1.35)**	ns	ns
Marital status	Married/cohabiting^c^	1.00	1.00	1.00
	Widowed/separated	**1.33 (1.22–1.45)**	**1.16 (1.09–1.24)**	**1.09 (1.03–1.16)**
	Never married	**1.24 (1.14–1.35)**	1.02 (0.95–1.09)	1.00 (0.94–1.08)
Education	More than 12 years^c^	1.00	1.00	1.00
	12 years	**1.54 (1.42–1.66)**	**1.17 (1.09–1.25)**	**1.23 (1.15–1.32)**
	Less than 12 years	**1.96 (1.79–2.14)**	**1.26 (1.16–1.36)**	**1.33 (1.25–1.42)**
Job status	With a job^c^	1.00		
	Without a job	**1.07 (1.01–1.14)**	ns	ns
Smoking status	Never smoker^c^	1.00	1.00	1.00
	Former smoker	1.04 (0.94–1.16)	1.05 (0.95–1.15)	1.00 (0.90–1.10)
	Current smoker	**1.57 (1.40–1.76)**	**1.18 (1.08–1.29)**	**1.17 (1.07–1.27)**
Self-rated oral health status	Satisfied^c^	1.00	1.00	1.00
	Not satisfied	**3.22 (2.73–3.80)**	**1.35 (1.24–1.47)**	**1.37 (1.26–1.48)**
Untreated dental caries	No^c^	1.00	1.00	1.00
	Yes	**1.86 (1.72–2.01)**	**1.30 (1.21–1.39)**	**1.32 (1.23–1.41)**
Income	High^c^	1.00	1.00	n/a
	Middle	**1.76 (1.57–2.00)**	**1.48 (1.31–1.67)**	n/a
	Near poor	**2.35 (2.07–2.66)**	**1.80 (1.58–2.05)**	n/a
	Poor	**2.44 (2.17–2.73)**	**1.69 (1.51–1.90)**	n/a
Wealth	High^c^	1.00	n/a	1.00
	Middle	**1.90 (1.74–2.08)**	n/a	**1.57 (1.44–1.71)**
	Low	**2.30 (2.05–2.58)**	n/a	**1.68 (1.52–1.85)**

*RR* Prevalence ratios using Poisson regression models, *CI* 95% Confidence Intervals, *n/a* Not Applicable, *ns* Not Significant

^a^ Parsimonious income model excluding country of birth and job status variables

^b^ Parsimonious wealth model excluding age, country of birth and job status variables

^c^ Reference Category

^d^ Data source – National Health and Nutrition Examination Survey (NHANES), 2011–2014

All bolded entries are statistcally significant

In the parsimonious income model, adults in the poor group were close to twice as likely to not have a dentist visit (RR = 1.69; 95% CI: 1.51–1.90) and those aged 20–44 years were 16% more likely to not have a dentist visit than those aged 65 years and over (RR = 1.16; 95% CI: 1.04–1.30). In the parsimonious wealth model, adults in the low wealth group were close to twice as likely to not have a dentist visit compared with those in the high wealth group (RR = 1.68; 95% CI: 1.52–1.85) and unlike the income model, there were no significant differences within age subgroups in the multivariable wealth model. This result shows that age and income were independently associated with not having dentist visits but the age association attenuated to non-significance in the presence of wealth.

The effect of being in either the Total Hispanic or Non-Hispanic Black group attenuated from the unadjusted to the parsimonious income and wealth models. This means that relative to being in the Non-Hispanic White group, the likelihood of those in either the Total Hispanic or Non-Hispanic Black group having had no dentist visits decreased in the presence of other socio-demographic factors. In contrast, there was positive attenuation for the Asian group, relative to the Non-Hispanic White group, for both the income and wealth models. The parsimonious models showed increased and significant associations between being in the Asian group and having no dentist visits which suggests possible offsetting sociodemographic effects in the unadjusted models. Overall, the risk indicators in both models were: being male; Hispanic; non-Hispanic black; widowed or separated; least educated; a current smoker; not satisfied with oral health; and having untreated caries. The income and wealth models were compared using the AIC. The AIC values for the parsimonious income and wealth models were 10,829 and 10,895 respectively. These results indicate that the income models are marginally better than the wealth models (the smaller the AIC value, the better the model).

**Fig. 1:**
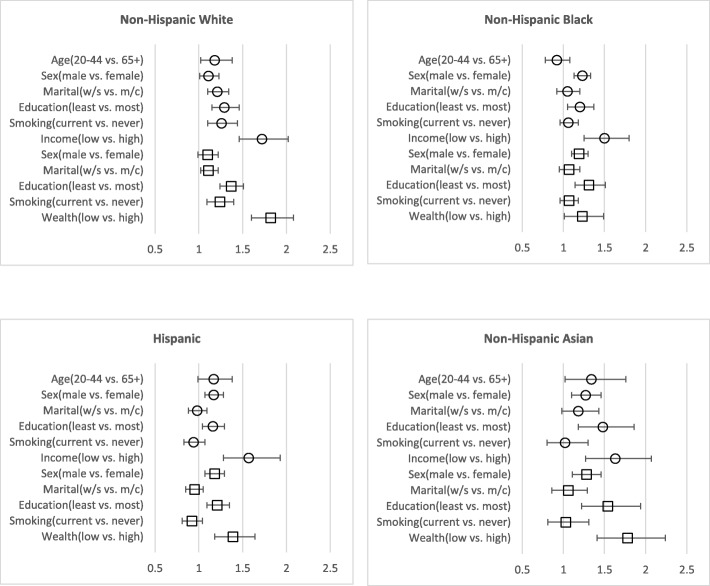
Income, wealth and other factors associated with not having a dentist visit in the past 12 months stratified by race/ethnicity among adults aged 20 years and over in the United States^+^. Each race/ethnicity plot contains two parsimonious multivariable regression models. Income represented by a circle (○). Wealth represented by a square (□). Measure of associations are prevalence ratios using Poisson regression models. Significant associations (*p* < 0.05) are those greater the one (in the figure) and do not cross the one line in the x-axis. Marital status – widowed/divorced/separated vs. married/cohabiting. [+] Data source – National Health and Nutrition Examination Survey (NHANES), 2011–2014

The analyses were stratified by race/ethnicity because of significant interactions. Figure 1 uses the parsimonious models to summarize the association (RRs). The figure presents income, wealth and other factors as binary variables to compare between the highest and lowest groups. In the income and wealth models, the poor and the low wealth respondents were more likely to not have a dentist visit in all four race/ethnic groups compared to the least poor and the high wealth groups. In the income model, age was a significant factor. Compared to participants aged 65 years and over, the non-Hispanic white and non-Hispanic Asian adults aged 20–44 years were more likely to not have a dentist visit, while the same association was not significant among non-Hispanic black and Hispanics. In the wealth model, age was not a significant factor in all race/ethnic groups.

## Discussion

This study is the first to describe both income and wealth as correlates of socioeconomic disparity in dentist visits among adults using NHANES data. Important differences were observed (especially in age groups) when each measurement was used. Age was not significant for wealth but significant for income. There were also significant interactions by race/ethnicity for income and wealth.

The overall prevalence of not having a dentist visit in the past 12 months among adults aged 20 years and over was 39%. This prevalence is consistent with other findings. For example, a report released in 2016 by the National Center for Health Statistics (NCHS) presented the proportion of adults aged 18 years and over without a dentist visit in 2014 at 38% [28]. However, studies using the Medical Expenditure Panel Survey (MEPS) data have reported higher percentages: 65% of adults aged 18–64 years did not see a dentist in 2013 [29]. Study design, sampling frame, reference periods, lead-in statements, question wording, and social desirability bias are some of the reasons for these different estimates [12].

Despite differences in these overall estimates of dentist visits, demographic and socioeconomic indicators such as sex, race/ethnicity and socioeconomic position have shown consistent associations across national US surveys. For example, persons in lower socioeconomic positions (commonly measured via income status and education) were significantly more likely than those in higher socioeconomic positions to not have a dentist visit in the past 12 months, as were non-Hispanic blacks; Hispanics were significantly more likely than non-Hispanic whites [12]. Similar findings were found in our study where participants in lower socioeconomic positions (measured by income status and wealth) were consistently more likely than those in higher socioeconomic positions to not have a dentist visit in the past 12 months. Other studies have also shown the same pattern in different contexts. A study among adults aged 50 years and over in 14 European countries reported higher rates of dental services utilization among the high-income group compared with the low-income group [30]. Income and education have also been reported as factors in not seeking oral health services in many other global contexts [31, 32].

Explanations for observed socioeconomic disparities have been put forward in the literature. The Commission on Social Determinants of Health (CSDH) produced a landmark report on the impact of social determinants of health (via unfair economic arrangements and poor social policies and programs) on the unequal distribution of health experiences, where health and illness invariably follow a social gradient [33]. Bartley [34] has also reviewed the four theories proposed to lie behind inequality in health and access to health care, namely behavioral, psychosocial, material, and life-course approaches [34]. Importantly, even well-defined material and neo-material resources (income, living conditions) and health behaviors (smoking, diet) do not fully explain health inequality, let alone the social gradient, which may be much more difficult to characterize. The persistent health inequality observed at all levels of the social gradient may be explained by the psychosocial model and the concept of buffering resources such as social capital, social support, social relations, social participation, and self-efficacy. Whatever the case, health inequalities are affected by a complex interaction of these pathways during a person’s life-course depending on the context and the population [34].

Our study shows that both income and wealth have strong associations with dentist visits, similar to findings in previous studies. For example, a study among US adults aged 51 years and older in 2008 showed the separate effects of income and wealth on dental utilization [35]. Likewise, a recent study among Japanese adults aged 50 to 75 years showed that both wealth-related and income-related inequalities in dental care use existed, with greater impact shown by wealth [36]. Our findings also show that results differ when measurements of income or wealth are used. For instance, age was significant in the income model but was not significant in the wealth model. Possible explanations for the observed differences may be that wealth reflects net accumulation of advantage and disadvantage over the life course while income reflects the direct and immediate impacts of a lack of resources. Moreover, without adjusting for wealth, participants aged 20–44 years were more significantly likely to not have a dentist visit but after adjusting for wealth and other sociodemographic factors the association was not significant. One possible explanation is that there are other cultural factors impacting on dental utilization.

There are some limitations. This is a cross-sectional study and causation cannot be inferred. We show that income and wealth are associated with dental visits. Furthermore, the study analyzed self-reported data, which might be subject to information and social desirability biases. For example, while respondents may report poor oral health, they could be misclassified based on clinical examination, or respondents might be more prone to provide socially acceptable responses or unable to correctly recall an event or to accurately evaluate their caries severity. Our outcome variable – not having a dentist visit in the past 12 months – may also capture a heterogeneous group of people who had various reasons for their answers to the question “about how long is it since you last visited a dentist?” Some people may have poor oral health because they had not visited the dentist in the past two or 3 years, whereas others may not have visited the dentist in the past 4 years, but have good oral health. We acknowledge that the outcome variable is not comprehensive but we are confident of its relevance, given that in the Healthy People 2020 objectives, one of the oral health objectives (OH-7) is to increase the proportion of children, adolescents, and adults who used the oral health care system in the past 12 months [6].

This is the first study of its kind to analyze wealth as a socioeconomic variable using NHANES data. We used two indicators - monthly family income and home ownership - to construct a wealth variable. Nevertheless, the results of our study should be interpreted with caution because capturing an individual’s wealth is a complex undertaking. One common method of measuring wealth is using Principal Component Analysis to generate weights indicative of household wealth. For this method to work, it requires gathering large amounts of information from respondents, such as ownership of material goods, savings, consumption of goods and services, etc. Unfortunately, due to data limitations we were not able to derive a wealth index using this method, but we think our approach may open doors for future surveys to incorporate more questions regarding household assets to create a wealth index that can give more precise measures of economic well-being. Lastly, we are unable to account for the role of environmental and psychosocial factors such as social capital, social support, geo-locality (urban/rural residence), as well as dental insurance and out of pocket payments. Studies have shown the impact of these determinants in oral health services utilization [37–39] but unfortunately, information on such factors was not collected in the NHANES.

## Conclusion

In the US, adults in lower socioeconomic positions (low wealth or income) are less likely to have annual dentist visits yet the socioeconomic patterning varies by age, race/ethnicity and other factors. This study showed that income may be a better measure of socioeconomic disparity than wealth, although wealth may well be a more suitable socioeconomic measure in older adult populations. There is clearly a need for further research into ways of more precisely measuring socioeconomic disparities in dentist visits in the US. Importantly, these findings strengthen the call to action regarding policy based on socioeconomic inequalities in oral health.

## Additional file

Additional file 1: Flowchart of participants in the study. (DOCX 27 kb)

## Abbreviations

AIC: Akaike Information Criterion
CI: 95% Confidence Interval
CSDH: Commission on Social Determinants of Health
ERB: Ethical Review Board
HHS: US Department of Health and Human Services
HP: Healthy people
MEC: Mobile Examination Centers
MEPS: Medical Expenditure Panel Survey
NCHS: National Center for Health Statistics
NHANES: National Health and Nutrition Examination Survey
ORs: Odds Ratios
PIR: Poverty Income Ratio
SE: Standard Errors
US: United States
