# Supplementary figures and images for: Income and wealth as correlates of socioeconomic disparity in dentist visits among adults aged 20 years and over in the United States, 2011–2014
Source: BMC Oral Health. 2018 Aug 23;18:147. doi: 10.1186/s12903-018-0613-4 (PMC6108097; doi:10.1186/s12903-018-0613-4)

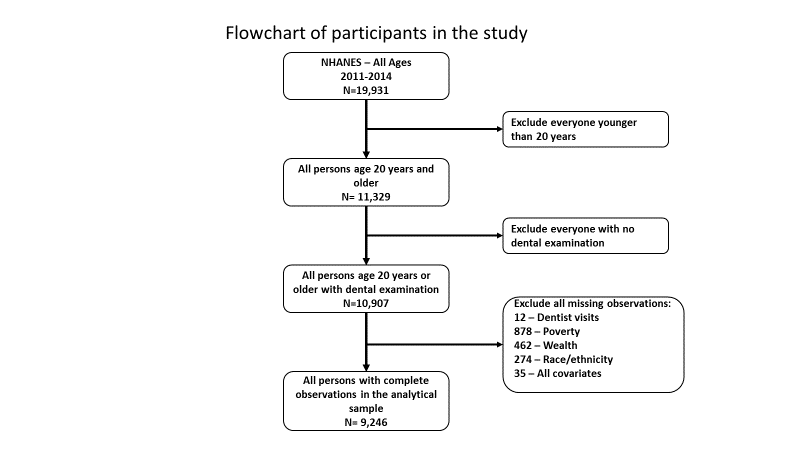

Supplement: Supplementary file 1 — Flowchart of participants in the study. (DOCX 27 kb) [file 12903_2018_613_MOESM1_ESM.docx]
